# The Agentic Era: Why Biopharma Must Embrace Artificial Intelligence That Acts, Not Just Informs

**DOI:** 10.7759/cureus.83390

**Published:** 2025-05-03

**Authors:** Shaheen E Lakhan

**Affiliations:** 1 Bioscience, Global Neuroscience Initiative Foundation, Miami, USA; 2 Neurology, A.T. Still University School of Osteopathic Medicine in Arizona, Mesa, USA; 3 Neurology, Morehouse School of Medicine, Atlanta, USA; 4 Neurology, Western University of Health Sciences, Pomona, USA

**Keywords:** agentic ai, artificial intelligence, autonomous systems, clinical trials, drug development, regulatory transformation

## Abstract

Agentic artificial intelligence (AI), characterized by its capacity for autonomous task execution rather than mere assistance, heralds a fundamental paradigm shift in biopharmaceutical operations. From accelerating discovery and streamlining development to revolutionizing commercialization and governance, agentic AI systems are poised to redefine the industry's core processes. This editorial explores the burgeoning role of agentic AI as a novel operating model for biopharma, highlighting its increasing relevance across critical functions, including drug development, clinical trials, regulatory affairs, and strategic decision-making. As regulatory frameworks adapt to accommodate AI-driven methodologies, agentic systems are positioned to supersede legacy infrastructures with self-directed, adaptive engines of innovation. The future of biopharma will be defined not only by groundbreaking molecular discoveries but also by the intelligent orchestration of these advancements through autonomous machines.

## Editorial

From smart tools to autonomous colleagues

Over the past decade, artificial intelligence (AI) has transitioned from a promising analytical tool to a critical enabler of enhanced efficiency within drug development [1]. Initial applications primarily supported discovery science, automated extensive literature reviews, and optimized patient recruitment for clinical trials. However, a transformative new class of AI systems has emerged, i.e., "agentic AI," capable of independently executing intricate and complex workflows. Unlike traditional machine learning models or dashboard-centric decision support systems that require human intervention, agentic AI operates with genuine autonomy. These sophisticated systems are engineered not simply to suggest actions, but to autonomously initiate and complete them, including generating comprehensive study protocols, simulating diverse trial arms, drafting detailed regulatory submissions, and triggering necessary supply chain adjustments - all without direct human initiation.

Drawing from my experience in spearheading innovation across various therapeutic areas, I have observed firsthand how these intelligent agents transcend the limitations of codified algorithms. They exhibit adaptive learning capabilities, maintain persistent memory of past interactions and contextual awareness, and possess the unique ability to seamlessly link tasks across disparate departments and geographical locations. For instance, one AI agent might autonomously generate a complete clinical trial protocol, while another simultaneously simulates various cost scenarios based on historical performance data. A third agent could then proactively initiate the pre-submission process with regulatory agencies. These are not isolated instances of automation; rather, they represent the emergence of digital colleagues operating with true enterprise-level intelligence and interconnectedness.

Agentic AI across the biopharma value chain

The transformative influence of agentic AI permeates the entire biopharmaceutical lifecycle. In the drug discovery phase, intelligent agents can propose novel drug candidates through sophisticated generative molecular design, efficiently cross-reference vast repositories of public and proprietary datasets, and rapidly simulate critical absorption, distribution, metabolism, excretion, and toxicity (ADMET) properties at unprecedented speeds. During development, clinical trials are increasingly shaped by AI systems capable of optimizing patient eligibility criteria, simulating complex patient flows, and generating adaptive trial designs in silico [2]. Agentic systems can now proactively predict potential patient dropout risks and issue real-time alerts to study teams, enabling the prevention of protocol deviations before they occur.

Regulatory functions are also undergoing a profound transformation. AI agents, meticulously trained on decades of regulatory submissions and reviewer feedback, are now capable of drafting comprehensive Common Technical Document (CTD) modules and even preparing detailed briefing materials for advisory committee meetings. As regulatory bodies themselves begin to adopt AI for efficient submission triage and sophisticated benefit-risk modeling, the future of regulatory oversight will likely evolve from traditional document review to more dynamic and continuous supervision.

The commercial and operational domains are experiencing parallel agentic transformation. AI systems can continuously monitor real-time formulary changes, analyze competitor strategies, and gauge key opinion leader sentiment to inform agile and adaptive launch strategies. Concurrently, intelligent manufacturing agents can respond dynamically to biologic stability data, optimize complex cold chain logistics, and orchestrate intricate facility-level workflows with minimal direct human oversight.

The recent, significant announcement by the FDA in 2025 to phase out mandatory animal testing for monoclonal antibodies and other drugs [3] underscores a growing regulatory acceptance of innovative alternative models, including simulation-first development strategies. This pivotal shift creates fertile ground for the widespread adoption of agentic systems capable of conducting predictive safety and efficacy analyses that can significantly reduce or even eliminate the traditional reliance on in vivo testing.

A compelling real-world example of this transformative shift is the strategic partnership between OpenAI (San Francisco, CA) and Moderna (Cambridge, MA) [4]. Moderna has successfully deployed sophisticated AI agents built on the advanced GPT-4 architecture to assist with a diverse range of critical tasks, from efficiently drafting complex regulatory documents to personalizing patient communications and synthesizing intricate datasets. Over 750 distinct internal use cases have already been identified, demonstrating the remarkable scalability of agentic systems in augmenting cognitive functions across legal, medical, manufacturing, and commercial teams. This is not merely about incremental automation; it represents a fundamental redefinition of how critical information flows and is processed throughout the entire organization.

Beyond acceleration: Redefining biopharma operations

While the biopharmaceutical industry often emphasizes the potential of AI for accelerating timelines and reducing costs, the fundamental and more profound value of agentic AI lies in its capacity to fundamentally reshape the very structure of how work is conducted. Agentic systems inherently break down traditional silos by seamlessly operating across diverse disciplines, integrating disparate knowledge domains in ways that human teams often struggle to achieve [5]. They significantly reduce the need for time-consuming coordination meetings, redundant review processes, and manual data reconciliations by autonomously executing complex, cross-functional tasks natively.

Consider a compelling scenario where an intelligent agent autonomously detects a critical shift in regulatory policy, proactively updates the relevant target product profile accordingly, meticulously simulates the potential impact of this change on key trial endpoints, and immediately alerts the relevant clinical team - all before a human analyst even notices the policy update. This transcends mere operational support; it represents true proactive strategic alignment driven by autonomous intelligence. Companies that strategically deploy these advanced systems are not simply operating faster; they are fundamentally becoming more intelligent, agile, and responsive organizations.

Equally transformative is the inevitable cultural shift that accompanies the widespread adoption of agentic AI. As autonomous systems increasingly execute critical decisions, organizational leadership must evolve its focus from granular micromanagement to defining overarching strategic goals, ensuring robust ethical governance, and aligning machine-driven outputs with core mission-driven objectives. This fundamentally reframes the role of executives from being primary overseers of activity to becoming strategic curators of organizational intelligence and orchestrators of human-machine synergy.

Barriers to adoption and essential ethical guardrails

Despite the considerable promise of agentic AI, its widespread adoption is not without significant challenges. Existing data silos, outdated legacy IT infrastructure, evolving regulatory uncertainties, and inherent institutional resistance to relinquishing traditional control mechanisms represent substantial barriers. Furthermore, the effective deployment of these sophisticated systems necessitates access to high-quality, meticulously curated training data, the development of robust explainability protocols to understand their decision-making processes, and the implementation of continuous validation strategies to maintain trust and reliability.

Of paramount importance are the critical ethical dimensions surrounding the deployment of autonomous AI in such a high-stakes industry. How do we effectively audit complex autonomous decisions? What robust safeguards can be implemented to prevent issues such as overfitting of training data or the propagation of AI "hallucinations" into regulated outputs? Without the establishment of deliberate and comprehensive governance frameworks, the inherent risks of automation bias, opaque algorithmic logic chains, and systemic errors within critical processes will inevitably grow.

Therefore, biopharmaceutical companies must proactively develop robust governance frameworks specifically designed to oversee machine-driven actions. This includes establishing comprehensive AI assurance protocols, maintaining detailed audit trails of autonomous decisions, and implementing clear and effective fail-safe escalation pathways for human intervention when necessary. This necessitates the formation of cross-disciplinary AI review boards, proactive engagement with regulatory agencies to shape future guidelines, and the development of transparent pipelines tracing the flow of information from initial training data to final autonomous action.

A call for agentic leadership

The successful navigation of this agentic era will require a new breed of leadership within biopharmaceutical organizations. This extends beyond the traditional role of chief digital officers to the emergence of chief agent officers (CAOs) - strategic leaders specifically tasked with aligning autonomous AI systems with overarching enterprise strategy and ensuring adherence to the highest ethical standards. These critical leaders must possess a unique blend of deep technical fluency and a strong commitment to human-centered oversight, ensuring that AI not only acts with speed and efficiency but also with wisdom, transparency, and a clear alignment with patient well-being.

Ultimately, the future of the biopharmaceutical industry belongs to those forward-thinking organizations that can effectively orchestrate the combined power of human and machine intelligence at scale. The next major therapeutic breakthrough may very well originate not solely from a traditional laboratory bench but from an intelligent AI agent autonomously simulating billions of potential molecular permutations in silico. When sophisticated AI acts intelligently and ethically on behalf of scientific progress, efficiency, and, most importantly, patients, the benefits will accrue to us all.
